# Endoplasmic Reticulum Stress, a Target for Drug Design and Drug Resistance in Parasitosis

**DOI:** 10.3389/fmicb.2021.670874

**Published:** 2021-05-31

**Authors:** Mei Peng, Fang Chen, Zhongdao Wu, Jia Shen

**Affiliations:** ^1^Department of Parasitology of Zhongshan School of Medicine, Sun Yat-sen University, Guangzhou, China; ^2^Key Laboratory of Tropical Disease Control (SYSU), Ministry of Education, Guangzhou, China; ^3^Provincial Engineering Technology Research Center for Biological Vector Control, Guangzhou, China; ^4^School of Medicine, South China University of Technology, Guangzhou, China

**Keywords:** endoplasmic reticulum stress, drug targets, parasite, parasitosis, drug resistance

## Abstract

Endoplasmic reticulum stress (ER stress) can be induced when cellular protein homeostasis is damaged, and cells can activate the unfolded protein response (UPR) to restore protein homeostasis or induce cell death to facilitate the survival of the whole system. Globally, parasites are a constant threat to human health and are therefore considered a serious public health problem. Parasitic infection can cause ER stress in host cells, and parasites also possess part or all of the UPR under ER stress conditions. In this review, we aim to clarify the role of ER stress pathways and related molecules in parasites for their survival and development, the pathogenesis of parasitosis in hosts, and the artemisinin resistance of *Plasmodium*, which provides some potential drug design targets to inhibit survival of parasites, relieves pathological damage of parasitosis, and solves the problem of artemisinin resistance.

## ER Stress and UPR

The endoplasmic reticulum (ER), a vital organelle in eukaryotic cells, is the site of synthesis and processing of membrane and secretory proteins, synthesis of lipids, and storage of Ca^2+^ (Dolai and Adak, 2014). Therefore, it is important to maintain ER homeostasis. Yet, many factors influence the protein homeostasis of ER, such as plasma cell differentiation (Gass et al., 2002), tunicamycin (Pahl and Baeuerle, 1995), and parasite infection (Galluzzi et al., 2017) which result in accumulated misfolded or unfolded proteins that exceed the folding capacity of ER and trigger endoplasmic reticulum stress (ER stress). Endoplasmic reticulum-associated degradation (ERAD) and unfolded protein response (UPR) are the two major quality control processes of ER stress (Bukau et al., 2006). UPR reduces the synthesis of proteins and eliminates misfolded proteins within the ER by increasing the expression of the ER chaperone proteins.

**FIGURE 1:**
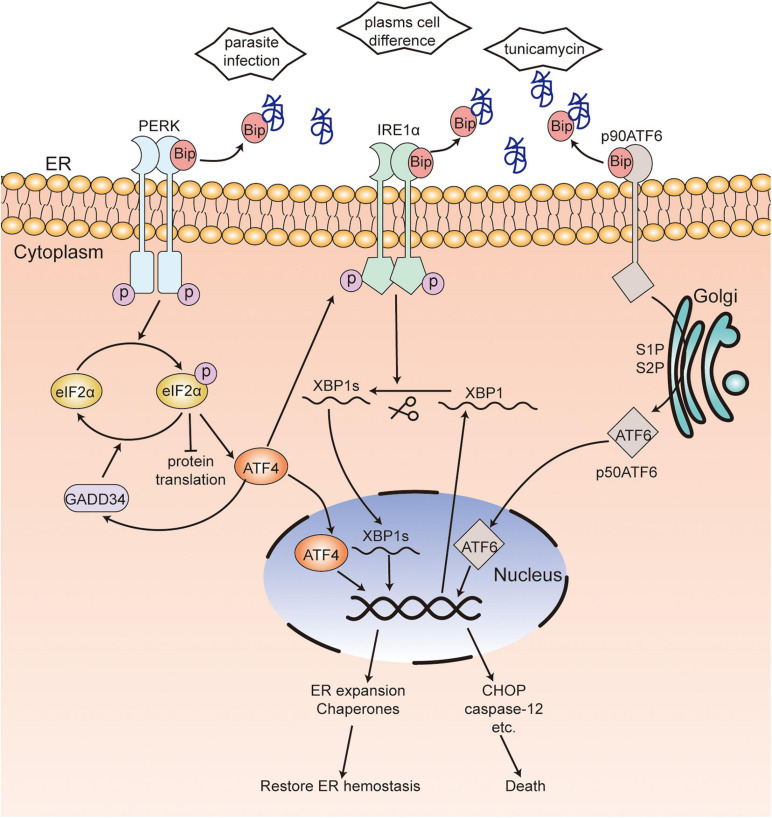
Unfolded protein response in mammalian cells. In unstressed cells, Bip binds to three transmembrane proteins PERK, IRE1α and ATF6. When ER stress occurs, Bip dissociates from these transmembrane proteins and binds to unfolded or misfolded proteins. Activated PERK, induced by oligomerization following dissociation from Bip, phosphorylates eIF2α, which reduces protein translation while upregulating the expression of ATF4. eIF2α phosphorylation can be dephosphorylated by GADD34. After dissociation from Bip, IRE1α is activated by forming IRE1α homodimers and incises XBP1 mRNA into XBP1s. Bip releases from ATF6, which leads to the translocation to Golgi and activation of ATF6. ATF4, XBP1s, and activated ATF6 enter the nucleus and activate the transcription of ER chaperones or various death effectors, which results in the restoration of ER hemostasis or cell death. [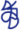], unfolded or misfolded proteins; PERK, protein kinase RNA-like ER kinase; IRE1, inositol-requiring kinase/endoribonuclease 1; eIF2α, α-subunit of eukaryotic translational initiation factor 2; ATF6, activating transcription factor 6; ATF4, activating transcription factor 4; XBP1, X box-binding protein 1; CHOP, C/EBP-homologous protein; Bip, immunoglobulin heavy chain binding protein; GADD34, DNA damage-inducible protein-34.

In mammalian cells, the UPR is mediated by three signaling pathways and activated by three ER-transmembrane proteins: inositol-requiring kinase/endoribonuclease 1 (IRE1), protein kinase RNA-like ER kinase (PERK), and activating transcription factor 6 (ATF6) (Figure 1; Hwang and Qi, 2018). Glucose-regulated proteins 78 (GRP78) (Bertolotti et al., 2000; Shen et al., 2002), also called immunoglobulin heavy chain binding protein (Bip) (Bertolotti et al., 2000; Shen et al., 2002), binds to these transmembrane proteins in unstressed cells, while it dissociates from them and binds to unfolded or misfolded proteins in stressed cells (Bertolotti et al., 2000; Shen et al., 2002; Grootjans et al., 2016). After dissociation from Bip, IRE1α will be activated by forming IRE1α homodimers and incise the transcription factor X box-binding protein 1 (XBP1) mRNA into spliced XBP1 (XBP1s) (Bertolotti et al., 2000; Calfon et al., 2002). The function of XBP1s is to maintain the ER function and response to UPR and regulate the expansion of the secretory apparatus (Acosta-Alvear et al., 2007). Activated PERK, induced by oligomerization and autophosphorylation following dissociation from Bip (Bertolotti et al., 2000), phosphorylates the α-subunit of eukaryotic translational initiation factor 2 (eIF2α) and attenuates protein translation, which will reduce the load of newly synthesized proteins within the ER while upregulating the expression of activating transcription factor 4 (ATF4). In addition, the phosphorylation of eIF2α can be dephosphorylated by growth arrest and DNA damage-inducible protein-34 (GADD34). Further, ATF4 is required for the transactivation of GADD34, which will promote the recovery of translation (Novoa et al., 2001; Ma and Hendershot, 2003). Under ER stress, ATF6 translocates from the ER to the Golgi apparatus (Shen et al., 2002). A 90-kDa protein ATF6 (p90ATF6) is converted to a 50-kDa protein ATF6 (p50ATF6, an active and mature form of ATF6) through the cleavage of Golgi-resident proteases—site 1 protease (S1P) and site 2 protease (S2P). P50ATF6 further activates the transcription of ER chaperone genes after entering the nucleus (Haze et al., 1999; Ron and Walter, 2007). Interactions among the three UPR pathways have been found, wherein ATF6 induces the transcription of XBP1 (Yoshida et al., 2001) and PERK-ATF4 upregulates the expression of IRE1α (Tsuru et al., 2016). These interactions will promote UPR to be stronger and more persistent in order to deal with various types of ER stress. Nevertheless, prolonged and severe ER stress can activate various cell death effectors such as BAK, BAX, caspase-12, C/EBP-homologous protein (CHOP), and GADD34 and induce cell death (Ron and Walter, 2007). The mechanism of UPR is evolutionary conservatism across eukaryotes.

## Parasitic Infection and UPR

Parasitosis, caused by parasitic infections, has been harmful to human health and economic development since very long and is still a major global public health problem. As is known, the effective control of parasitic diseases is mainly dependent on the application of parasitic drugs and disruption of the pathogen’s life cycle, such as praziquantel and artemisinin. Unfortunately, drug resistance of parasitic drugs has been reported in recent years (Fallon and Doenhoff, 1994; Dondorp et al., 2009). Therefore, new anti-parasitic drugs including those to alleviate the pathology of the host caused by parasite infection and those to kill parasites should be identified urgently, along with determination of the mechanisms of drug resistance.

When parasites infect the host and obtain nutrients, they will perturb ER homeostasis and induce ER stress and UPR of the host. On the one hand, the induced ER stress of the host is beneficial to the survival and infection of the parasites. For instance, it has been reported that *Plasmodium berghei* infection induced ER stress of hepatocytes and activated UPR through the XBP1 and cAMP responsive element-binding protein (CREBH, a hepatocyte specific UPR mediator) pathways, which contributed to the infection of *Plasmodium* by providing phosphatidylcholine and regulating iron level (Inacio et al., 2015). In addition, *Leishmania* infection induced ER stress of the host to facilitate infection through the PERK-eIF2α-ATF4 and IRE1-XBP1 pathways (Dias-Teixeira et al., 2016, 2017; Galluzzi et al., 2016; Abhishek et al., 2018). And *Toxoplasma* triggered the UPR in host cells, which affected calcium release from ER, can enhance host cell migration and dissemination of the parasite to host organs (Augusto et al., 2020). However, Poncet et al. (2021) have showed that the IRE1a/XBP1s branch of the UPR was a key regulator of host defense upon *Toxoplasma gondii* infection, that mice deficient for IRE1a and XBP1 in DCs displayed a severe susceptibility to *T. gondii* infection, which indicates that the UPR induced by parasites also plays an important role in host immune defense. Anyhow, on the other hand, excessive ER stress and UPR will cause severe pathological damage to the host. Yu et al. (2014) found that the levels of GRP78, CHOP, cleaved caspase-12, and phosphorylated-JNK in the intestine of *Trichinella spiralis*-infected mice were significantly upregulated, which indicated that the ER stress-induced apoptotic pathway participated in intestinal lesions caused by *T. spiralis* infection. Thus, inhibition of excessive UPR in the host may be a therapeutic target to alleviate the pathological symptoms.

Additionally, the parasites can sense ER stress and either induce UPR to facilitate their survival when attacked by the host immune system or adapt to the host environment (such as changes in pH and temperature, oxidative stress, nutrient deficiency) (Zuzarte-Luís and Mota, 2018). Therefore, the UPR signaling pathway may be a potential target for inhibiting the survival and development of parasites.

## The UPR in the Host May Be a Therapeutic Target for Relieving Pathological Damage of Parasitosis

Different parasitic infections result in different pathological damage to different host tissues and organs. Nowadays, increasing reports show that ER stress and UPR play an important role in the development of pathology of parasitosis (Anand and Babu, 2013; Ayyappan et al., 2019).

### Plasmodium

*Plasmodium* spp., which are the causative agents of malaria, are obligate intracellular protozoan parasites. Anand and Babu (2013) reported that experimental cerebral malaria (ECM), caused by *P. berghei* ANKA (PbA) infection, was related to ER stress. They found that PbA infection-induced ER stress could cause the apoptosis of neuronal cells in mice by activating the three branches of UPR—PERK-eIF2α-ATF4/GADD34, IRE1-XBP1s, and ATF6—along with upregulating the levels of CHOP, cleaved caspase-3 and caspase-12 and downregulating the expression of Bip, calreticulin, and calnexin.

### Trypanosome

*Trypanosome cruzi* is the causative pathogen of Chagas disease in humans. Reportedly, the trypomastigotes of *T. cruzi* infection could induce ER stress in the heart of mice, with an increase in the levels of Bip, PERK, eIF2α, ATF4, and CHOP, thereby causing damage to the host. Interestingly, 2-aminopurine (2-APB, an ER stress inhibitor) treatment could alleviate the pathological damage to the heart by decreasing the phosphorylation of eIF2α and its downstream signaling. Therefore, this indicates that inhibition of ER stress may be a therapeutic target for cardiomyopathy in Chagas patients (Ayyappan et al., 2019).

### Toxoplasma

*Toxoplasma* is an obligate intracellular parasite and opportunistic pathogenic parasite (Sullivan et al., 2004). *Toxoplasma* encephalitis is the most serious outcome of toxoplasmosis, which may be fatal to immunocompromised individuals. Some studies have found that *Toxoplasma* encephalitis was related to ER stress. It has been reported that the tachyzoites of *T. gondii* RH strain and TgCtwh3 (a representative Chinese 1 *Toxoplasma* strain) induced apoptosis of neural stem cells and neural stem cell line C17.2 by activating CHOP, caspase-12, and JNK (Wang et al., 2014; Zhou et al., 2015). Pretreatment with tauroursodeoxycholic acid (TUDCA, an ER stress inhibitor) and Z-ATAD-FMK (a caspase-12 inhibitor) led to the inhibition of apoptosis (Wang et al., 2014; Zhou et al., 2015), which suggested that neural stem cell apoptosis induced by both TgCtwh3 and RH strain infection was dependent on the ER stress pathway, and ER stress inhibitors could be used to alleviate *Toxoplasma* encephalitis. In addition, Wan et al. (2015) showed that virulence factor rhoptry protein 18 (ROP18) secreted by *T. gondii* was involved in nerve cell apoptosis *via* the ER stress pathway, characterized by an increase in the expression of cleaved caspase-12, CHOP, and cleaved caspase-3. Ran et al. further indicated that ROP18 induced apoptosis of neural cells by phosphorylating reticulon 1-C [RTN1-C, a protein localized in the ER that is preferentially expressed in the neural cells of the central nervous system (CNS) at Ser7/134 and Thr4/8/118], which led to the acetylation of GRP78 and induced ER stress (An et al., 2018). These results suggest that inhibition of ROP18 of *T. gondii* can be used as a drug target for the treatment of *Toxoplasma* encephalitis to inhibit the ER stress-induced apoptosis of host cells.

### Schistosoma japonicum

*Schistosoma japonicum* is the causative agent of schistosomiasis. The pathogenic mechanism of schistosomiasis is primarily attributed to egg-induced hepatic granuloma and fibrosis and cirrhosis (Yu et al., 2016; Duan et al., 2019). Duan et al. (2019) showed that the level of CHOP, a vital factor in the ER stress-mediated apoptosis pathway, was significantly increased in mice at 6 and 10 weeks following infection with *S. japonicum*. The study indicated that ER stress may be involved in *S. japonicum* infection-induced hepatic fibrosis. Moreover, Yu et al. (2016) showed that treatment with taurine, an inhibitor of ER stress, significantly suppressed the egg-induced hepatic granuloma and alleviated hepatic fibrosis in mice at 8 weeks post-infection, along with marked reduction of the expression of GRP78. Therefore, ER stress inhibitors may be a therapeutic drug for hepatic fibrosis.

**FIGURE 2:**
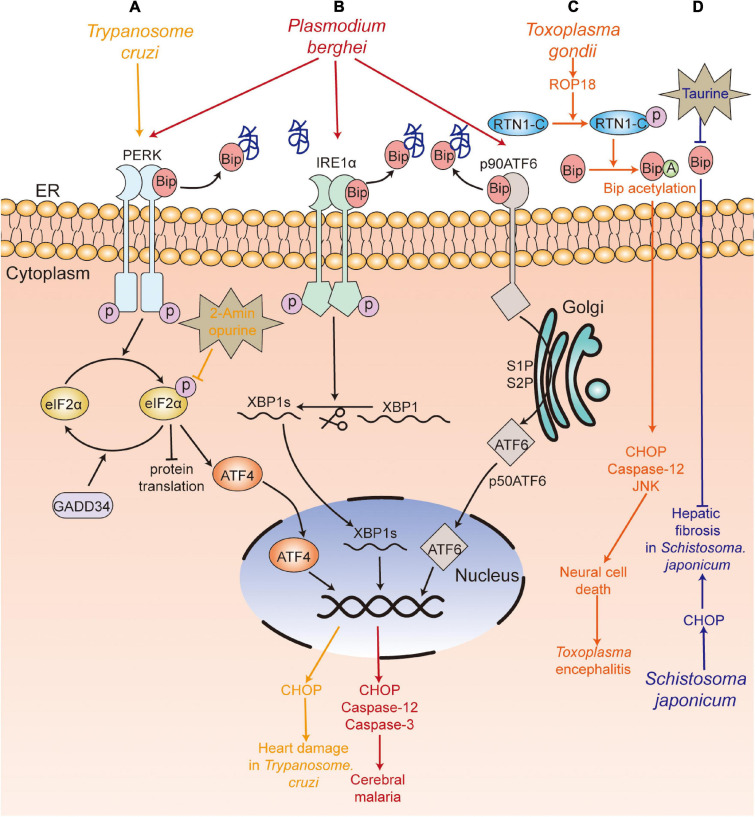
UPR participates in the pathological damage caused by parasite infection. **(A)** *T. Cruzi* (yellow) infection caused heart damage, with upregulation of PERK-eIF2α-ATF4-CHOP pathway, and 2-aminopurine treatment alleviated the heart’s pathological damage. **(B)** *P. berghei* (red) infection induced neuronal cell death and caused experimental cerebral malaria by activating the three branches of the UPR (PERK-eIF2α-ATF4/GADD34, IRE1-XBP1s, ATF6). **(C)** Rhoptry protein 18 (ROP18) of *T. gondii* (orange) phosphorylated reticulon 1-C (RTN1-C), which led to the acetylation of GRP78 and further upregulated the expression of cleaved caspase-12, CHOP, cleaved caspase-3, and induced the apoptosis of neural cells. **(D)** *S. japonicum* (blue) infection led to increased levels of CHOP, which was involved in hepatic fibrosis, and the treatment with taurine suppressed the egg-induced hepatic granuloma and fibrosis. PERK, protein kinase RNA-like ER kinase; IRE1, inositol-requiring kinase/endoribonuclease 1; eIF2α, α-subunit of eukaryotic translational initiation factor 2; ATF6, activating transcription factor 6; ATF4, activating transcription factor 4; XBP1, X box-binding protein 1; CHOP, C/EBP-homologous protein; Bip, immunoglobulin heavy chain binding protein;GADD34, DNA damage-inducible protein-34.

The summary of ER stress in hosts caused by parasitic infection is shown in Figure 2. Therefore, the UPR signaling pathway may be a therapeutic target to alleviate pathological symptoms.

## The UPR in Parasites Suggests Potential Drug Targets for Inhibiting the Survival and Development of Parasites

Parasites can sense ER stress and induce UPR of themselves to facilitate their survival and development. Different parasites may have different components of ER stress pathway.

### Plasmodium

*Plasmodium* has a complicated life cycle, including the merozoite, ring, trophozoite, schizont, and gametophyte stages in humans and the ookinete and sporozoite stages in mosquitoes. Chaubey et al. (2014) showed that *Plasmodium falciparum* lacked the orthologs of XBP1, IRE1, ATF6, and ATF4, and only retained the PERK-eIF2α pathway to regulate translation under ER stress. Three eIF2α kinases have been identified, namely IK1, IK2, and PK4 (eIF2α kinase of *Plasmodium* (Möhrle et al., 1997), a PERK homolog of mammals) (Ward et al., 2004). It has been reported that increased phosphorylation of eIF2α leads to reduced levels of protein translation, which is associated with the formation of *P. falciparum* gametophytes and the conversion of the *P. berghei* gametophytes into ookinetes when treated with dithiothreitol (DTT) (Chaubey et al., 2014; Duran-Bedolla et al., 2017). In addition, Zhang et al. (2012) have shown that PK4 was involved in the invasion of new red blood cells of merozoite-containing schizonts and the gametocyte infecting *Anopheles* mosquitoes. The inhibition of PK4 of *P. berghei* by generating a PK4 conditional mutant (PbPK4cKO) would alleviate the symptoms of malaria and inhibit disease transmission. Another study indicated that treatment of GSK2606414 (a small molecule inhibitor of PERK (Axten et al., 2012), which specifically inhibits PK4 instead of IK1 and IK2 *in vitro*) could block the transformation of *P. falciparum* from trophozoites to schizonts (Zhang et al., 2017). The transformation between different forms increased the ability of translational regulation of *Plasmodium*. In addition, Chen et al. (2018) reported that apoptozole, a novel chemical scaffold, was lethal to the chloroquine-sensitive and chloroquine -resistant *P. falciparum* parasite strains by inhibiting GRP78 function *in vitro*. Compared to human GRP78, *P. falciparum* GRP78 showed a lower affinity to the endogenous ligands, ADP and ATP, which indicated that the competitive inhibitors of GRP78 can be investigated for *P. falciparum* control (Chen et al., 2018).

According to the above mentioned studies, it appears that the PK4-eIF2α pathway plays an important role in both morphological transformation and host transmission in *Plasmodium*. Thus, PK4 inhibition would inhibit the development of *Plasmodium*, which implies that PK4 inhibitors may be a potential target in malaria treatment. However, Bridgford et al. (2018) found that dihydroartemisinin (DHA) increased the toxicity to *Plasmodium* by prolonging PK4 activation and eIF2α phosphorylation. Therefore, appropriate ER stress is beneficial to the development of *Plasmodium*, while excessive ER stress would be lethal to the parasites.

### Leishmania

*Leishmania* is the pathogen causing Leishmaniasis and has two forms—promastigote and amastigote. Gosline et al. (2011) proved that *Leishmania* lacked a transcriptional regulation response to UPR, and only retained the translational regulation in ER stress. They also showed an increased level of phosphorylation of eIF2α in *L. donovani* after treatment of DTT (Gosline et al., 2011). Moreover, Chow et al. (2011) found that the PERK homolog of *Leishmania* largely colocalized with Bip in ER, which can phosphorylate eIF2α at threonine 166. They further confirmed that PERK-dependent eIF2α phosphorylation was vital for *Leishmania* to switch from the promastigote to amastigote form *in vitro* (Chow et al., 2011). Unlike host macrophages having intact UPR pathway, the mere presence of the PERK pathway in *L. donovani* promoted the parasite’s susceptibility to DTT-induced ER stress (Gosline et al., 2011), which suggests that inhibition of the PERK pathway and induction of ER stress in *Leishmania* are both potential targets to kill the parasite. Dolai et al. (2011) proved that tunicamycin treatment induced apoptosis of *Leishmania major*, with an increase in the level of Bip.

### Trypanosome

*Trypanosome brucei* is a protozoan parasite that cycles between the tsetse fly (procyclic form) and mammalian host (blood stream form), which causes African sleeping sickness in humans and nagana in livestock (Zhang et al., 2019). Goldshmidt et al. (2010) reported that the expression of Bip of *T. brucei* was increased in both procyclic and blood stream forms in DTT-induced ER stress, and irrecoverable ER stress could induce spliced leader RNA silencing pathway (SLS pathway, a unique process in *T. brucei*), which may accelerate programmed cell death (PCD). Besides, Messias Sandes et al. (2019) showed that both DTT and tunicamycin could induce PCD in *T. cruzi*.

There were three putative eIF2α kinases (TbeIF2K1-K3) in *T. brucei*, though its genome lacked the homologs of IRE1/XBP1. It was reported that TbeIF2K2, a transmembrane glycoprotein expressed both in the procyclic and bloodstream forms of *Trypanosome* (Moraes et al., 2007), shared no similar sequence with known eIF2 kinases of mammals and was localized to the flagellar pocket, where endocytosis and exocytosis occur, and all proteins were transported from the flagellar pocket to the cell membrane (Gull, 2003). Therefore, the localization of TbeIF2K2 indicated that it could sense proteins and regulate protein synthesis near the flagellar pocket of the *Trypanosome* (Moraes et al., 2007), which suggests that TbeIF2K2 may be a good drug target to destroy *T. brucei*. In addition, Hope et al. (2014) showed that SEC63 (a factor participating in protein translocation machinery in ER) silence-induced ER stress could activate PK3 (TbeIF2K3) and trigger the release of PK3 from the ER to nucleus in the procyclic form of *T. brucei*. The deletion of PK3 reduced the death of *T. brucei* in SEC63 silence-induced ER stress, which suggests that PK3 is required for ER stress-induced PCD. Thus, the results indicate that TbeIF2K2 and TbeIF2K3 could be potential drug targets to eliminate *T. brucei*.

The PERK-eIF2α pathway is also involved in the form transformation of *T. cruzi* at different developmental stages. Tonelli et al. (2011) reported that the differentiation of non-infective epimastigotes into infective metacyclic trypomastigotes in *T. cruzi* requires the phosphorylation of Tc-eIF2α.

In conclusion, the results show that eIF2α phosphorylation plays an important role in the survival and development of *Trypanosome*, while excessive ER stress induced by DTT or tunicamycin can lead to the death of *Trypanosome*.

### Toxoplasma

*Toxoplasma* shows two forms in the human host: tachyzoite (a rapidly growing form) and bradyzoite (a quiescent cyst form) (Black and Boothroyd, 2000; Sullivan et al., 2004). It has been reported that *T. gondii* lacked the homologs of IRE1 and ATF6 (Joyce et al., 2013), while it possessed four TgIF2α kinases, namely TgIF2K-A, TgIF2K-B, TgIF2K-C, and TgIF2K-D (Narasimhan et al., 2008; Konrad et al., 2014). Narasimhan et al. (2008) showed that only TgIF2K-A was a transmembrane protein localized in the ER and bonded to Bip under unstressed conditions. When ER stress occurred, the binding of Bip to TgIF2K-A was reduced, similar to the binding of BiP to PERK in mammals, which suggests that part of the UPR was conserved in *T. gondii* (Narasimhan et al., 2008).

ER stress is also involved in the differentiation of *Toxoplasma.* Narasimhan et al. (2008) reported that the phosphorylation of TgIF2α induced by tunicamycin treatment resulted in the differentiation of *T. gondii* from tachyzoite to bradyzoite cysts. Treatment with salubrinal, an inhibitor of eIF2α dephosphorylation, could also induce the differentiation of bradyzoite cysts, which indicated that TgIF2α phosphorylation was involved in the differentiation of bradyzoite cysts (Narasimhan et al., 2008). Cyst formation is a good way to escape from the host’s immune attack. Therefore, the formation of bradyzoite cysts induced by TgIF2α phosphorylation promotes the survival of *T. gondii* under stressful conditions. Similar results were confirmed by Joyce et al. (2013). Besides, Joyce et al. (2010) reported that the TgIF2α mutant strain of *Toxoplasma* (i.e., TgIF2α-S71A, which cannot be phosphorylated) showed a lower virulence to the host cell, a lower survival rate and a slower transmitting speed, compared with the control strain of *Toxoplasma*. Moreover, Augusto et al. (2018) showed that specific inhibition of TgIF2K-A with GSK2606414 could inhibit the lytic cycle of tachyzoites, including attachment/invasion, replication, egress, and differentiation, which prolonged the survival time of mice with acute toxoplasmosis at a lethal dose of 100 RH strain tachyzoites. Interestingly, GSK2606414 did not show apparent detrimental effects on the host cell though with a high concentration *in vitro*. Therefore, the results suggest that TgIF2K-A and TgIF2α can be used as drug targets to inhibit *Toxoplasma* survival.

However, DTT treatment and stearoyl-coenzyme A (CoA) desaturase (SCD) accumulation at the ER could trigger ER stress with increasing phosphorylation of TgIF2α and mediated the apoptosis or autophagy of *T. gondii* (Nguyen et al., 2017; Hao et al., 2019). Therefore, although the TgIF2K-A/TgIF2α pathway plays a protective role in *T. gondii* under stress conditions, severe disruption of ER homeostasis can lead to the death of *T. gondii*.

### Entamoeba histolytica

*Entamoeba histolytica* infection, caused by ingestion of cysts in contaminated water and food, usually induces amoebic dysentery and liver abscesses in humans (Pineda and Perdomo, 2017). Santi-Rocca et al. (2012) found that no genes encoded the orthologs of PERK and ATF6 in *E. histolytica* amoeba, while the expression of gene encoding eIF2α was upregulated upon treatment with nitric oxide (NO). Hendrick et al. (2016) showed that eIF2α could be phosphorylated at serine-59 in *E. histolytica*, with a decrease in translation levels during long-term serum starvation, long-term heat shock, and oxidative stress instead of short-term serum starvation, short-term heat shock, and glucose deprivation, and the viability of EheIF2α-S59D (a phosphomimetic variant of eIF2α) was significantly increased during long-term serum starvation. This study suggests that EheIF2α phosphorylation promotes the survival of *E. histolytica* under stress conditions. DTT treatment can also induce distinct fragmentation of ER and phosphorylation of EheIF2α, while treatment with SNP and DPTA-NON-Oate (NO donors) did not induce phosphorylation of EheIF2α (Walters et al., 2019). Besides, Kumari et al. (2018) identified the ortholog of IRE1 in *E. histolytica* (EhIre1) and reported that treatment with tunicamycin resulted in the upregulation of EhIre1. In addition, the level of eIF2α phosphorylation was increased during encystation of *Entamoeba invadens*, but whether eIF2α is necessary for encystation still needs further investigation (Hendrick et al., 2016).

### Echinococcus granulosus

*Echinococcus granulosus* is the causative cestode of hydatidosis or cystic echinococcosis (CE) and is a worldwide zoonotic infection that affects many organs in human and mammals (Loos et al., 2018). Nicolao et al. (2017) have identified the ortholog of IRE2, XBP1, and ATF6 in the genome of *E. granulosus*, but the ortholog of PERK/ATF4 was not found. Treatment with bortezomib (a proteasome inhibitor) led to lower viability of *E. granulosus* in the larval stage *in vitro* than that in the control group, with an increase of EgGRP78 and EgIRE2/EgXBP1 mRNA levels in protoscoleces; however, no changes were found in the metacestodes (Nicolao et al., 2017). Another study also showed that arsenic trioxide (As_2_O_3_) could disturb the intracellular Ca^2+^ homeostasis and activated ER stress-related apoptosis of protoscoleces *in vitro*, with an increase in the expression of GRP78, caspase-3, and caspase-12 (Li et al., 2018). These studies show that the induction of ER stress can lead to the apoptosis of protoscoleces *in vitro*.

**FIGURE 3:**
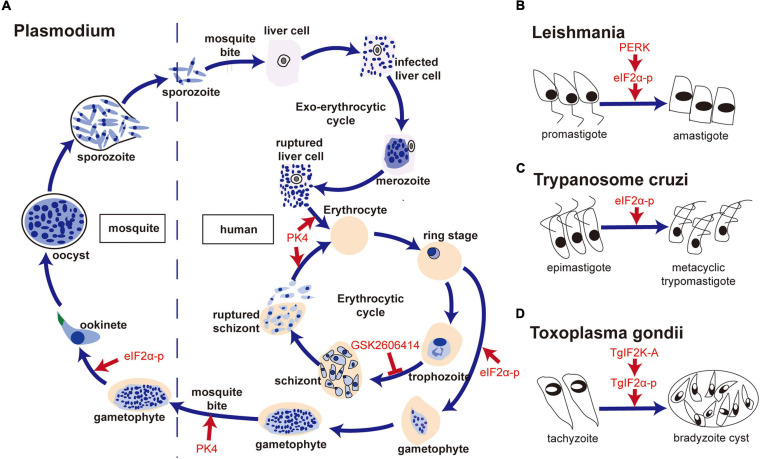
The PERK-eIF2α pathway is involved in forms transformation of parasites. **(A)** PK4-eIF2α was associated with the formation of gametophytes, conversion of *Plasmodium* gametophytes into ookinetes, invasion of new red blood cells by merozoite-containing schizonts and the infection of *Anopheles* mosquitoes by gametocytes; **(B)** PERK- eIF2α phosphorylation was vital for the switch from promastigote to amastigote in *Leishmania*; **(C)** Tc-eIF2α phosphorylation was required for the differentiation of non-infective epimastigotes into infective metacyclic trypomastigotes of *T. cruzi*; **(D)** TgIF2K-A/TgIF2α phosphorylation was related to bradyzoite cyst differentiation in *T. gondii*.

**FIGURE 4:**
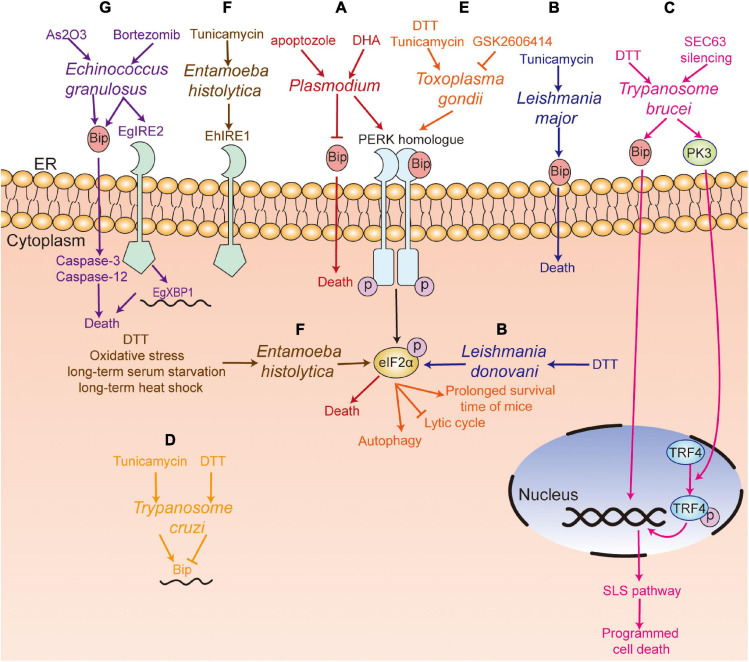
UPR pathway in parasites. **(A)** *Plasmodium* spp. (red) with the PERK homolog; apoptozole was lethal to the chloroquine-sensitive and chloroquine-resistant *P. falciparum* parasite strains by inhibiting GRP78 function *in vitro*, and DHA treatment could induce the death of *P. falciparum* through the PERK-eIF2α pathway; **(B)** Tunicamycin treatment induced the death of *L. major* (blue) with an increased level of Bip, and DTT treatment upregulated the phosphorylation of eIF2α in *L. donovani*; **(C)** DTT treatment induced programmed cell death. SEC63 silencing activated PK3, which further induced programmed cell death by phosphorylating TRF4 and inducing the SLS pathway in *T. brucei* (pink); **(D)** Tunicamycin treatment increased the level of Bip mRNA, while DTT treatment decreased the level of Bip in *T. Cruzi* (yellow); **(E)** GSK2606414 inhibited the lytic cycle of tachyzoites, including attachment/invasion, replication, egress, and prolonged the survival time of infected mice. DTT treatment mediated the apoptosis or autophagy of *T. gondii* (orange) by increasing the phosphorylation of TgIF2α; **(F)** DTT treatment, long-term serum starvation, long-term heat shock and oxidative stress induced the phosphorylation of eIF2α, and the ortholog of IRE1 in *E. histolytica* (EhIre1) (gray) was identified; **(G)** Bortezomib treatment induced the death of *E. granulosus* (purple) with an increase of EgGRP78 and EgIRE2/EgXBP1 mRNA. Arsenic trioxide treatment induced the death of *E. granulosus* (purple) with upregulation of the expression of GRP78, caspase-3, and caspase-12 in protoscoleces. PERK, protein kinase RNA-like ER kinase; IRE1, inositol-requiring kinase/endoribonuclease 1; XBP1, X box-binding protein 1; Bip, immunoglobulin heavy chain binding protein; eIF2α, α-subunit of eukaryotic translational initiation factor 2; TRF4, TBP-related factor 4; SLS pathway, spliced leader RNA silencing pathway; DTT, dithiothreitol; DHA, dihydroartemisinin.

In sum, the components of the UPR response such as the PERK-eIF2α pathway of some parasites (Figures 3, 4), including *Plasmodium*, *Leishmania*, *Trypanosome*, *Toxoplasma*, and *E. histolytica*, play an important role in their survival and development. However, excessive ER stress could induce the death of parasites such as *Plasmodium*, *Leishmania*, *Trypanosome*, *Toxoplasma*, and *E. granulosus* (Figure 4). Considering the toxicity of commonly used ER stress inducers such as DTT and tunicamycin, it is difficult to use them to kill parasites *in vivo*. For those parasites that are more sensitive to ER stress inducers than their hosts, it is necessary to explore the appropriate concentration of these inducers. TUDCA, a bile salt and chemical chaperone used to treat biliary cirrhosis clinically (Lazaridis et al., 2001), partially inhibits ER stress by lowering the levels of PERK, Bip (Malo et al., 2010; Liu et al., 2015; Li et al., 2019). Thus, TUDCA may be an alternative therapy for parasitosis.

Endoplasmic reticulum-associated degradation is another way for maintaining ER homeostasis, which can degrade misfolded protein (Hwang and Qi, 2018). And ERAD also exists in parasites, such as trypanosomes (Tiengwe et al., 2016). In addition, some apicomplexan parasites, including *P. falciparum*, *T. gondii* and *cryptosporidium*, harbor an apicoplast, which is important for parasite survival (Agrawal et al., 2013). Reportedly, ERAD components were associated with importing the apicoplast protein, and lose of ERAD components would lead to the death of parasites (Agrawal et al., 2009; Spork et al., 2009). Thus, apicoplast is a potential anti-parasitic drug target. Ubiquitin-dependent ERAD is essential for the survival of *Plasmodium* (Chung et al., 2012). Harbut et al. found that the inhibitors of signal peptide peptidase (SPP, a protein of ERAD) was lethal to *P. falciparum* (Harbut et al., 2012). It was reported that NITD731, a SPP inhibitor, was effective against *T. cruzi* and *T. gondii*, and it showed no toxicity to human cell lines (Harbut et al., 2012). Above studies further showed that parasites were much more sensitive to the disruption of protein homeostasis. Thus, inhibition of the two key quality-control mechanisms, UPR and ERAD, may be a potential way for parasites control.

## UPR in Parasites Is Involved in Artemisinin Resistance and Recrudescence of *Plasmodium*

As is well known, UPR can restore ER homeostasis. Therefore, when parasites are exposed to external risk factors such as drugs, they are capable of restoring their own homeostasis by inducing ER stress and activating UPR; hence, it is not surprising that ER stress and UPR are involved in the mechanism of drug resistance.

Artemisinin-based combination therapies (ACTs) are efficient frontline drugs to treat malaria. However, since artemisinin resistance was first discovered *in vivo* in western Cambodia (Dondorp et al., 2009), it gradually become a great challenge in malaria treatment. In recent years, many researches have focused on the mechanism of artemisinin resistance, and some of them have suggested that UPR is an important mechanism for artemisinin resistance. By analyzing 1,043 *P. falciparum* samples isolated from the peripheral blood of patients with acute malaria, Mok et al. (2015) found that artemisinin-resistant parasites exhibited decelerated development in the early ring stage and the expression of two molecular chaperone complexes of UPR were upregulated, such as *Plasmodium* reactive oxidative stress complex (PROSC, BiP belonging to the family) (Haldar et al., 2018) and TCP-1 ring complex (TRiC) (Mok et al., 2015). Thus, they speculated that the decelerated development of artemisinin-resistant *P. falciparum* may be associated with the upregulation of their UPR, which as a proteostatic mechanism that can repair the artemisinin induced impaired protein and reduce artemisinin-induced toxic proteopathy (Mok et al., 2015). Souvik et al. further clarified that the amplified phosphatidylinositol-3-phosphate (PI3P) tubules/vesicles in the parasite’s ER in infected red cells extensively spread the proteostatic capacity of UPR, which may neutralize artemisinin’s toxic proteopathy and participate in artemisinin resistance (Bhattacharjee et al., 2018). Therefore, ER stress inhibitors or PI3P tubules/vesicles inhibitors may be used in patients with artemisinin resistance.

Zhang et al. (2017) studied the relationship between PK4-eIF2α pathway and recrudescence of *Plasmodium* and found that treatment of ARTs could activate the phosphorylation of PK4-eIF2α and promote latency in the ring stage. Treatment with salubrinal significantly increased the recrudescence rate, while the PK4 inhibitor GSK2606414 abolished recrudescence after ARTs treatment in *P. berghei*-infected mice. Furthermore, they also showed that eIF2α phosphorylation was only observed in the young ring stage of Dd2^*C580Y*^ but not in Dd2, an ART-sensitive and chloroquine-resistant *Plasmodium* line. This study indicated that the recrudescence of *Plasmodium* was related to the activation of PK4 and phosphorylation of eIF2α following ART treatment. The results show that artemisinin can be combined with PK4 inhibitor to prevent the recurrence of *Plasmodium*.

## Conclusion and Perspectives

Parasitic infection-induced pathological damage in hosts largely depends on ER stress. Therefore, inhibition of ER stress in hosts can be an effective treatment approach for parasitic diseases. In addition, considering that ER stress of parasites participates in their survival, development, and infection, the components or molecules of ER stress of parasites may be used as drug targets to kill or inhibit the development of parasites. The ER stress components or molecules that may be potential targets for the treatment of parasitic diseases are summarized in Table 1. Chemical chaperones TUDCA, trehalose, and 4-phenylbutyrate (4-PBA) have been used to reduce ER stress (Hetz et al., 2013), and they may be available for clinical application. Besides, clinical trials have shown that TUDCA therapy improved the sensitivity of insulin in the liver and muscle of insulin-resistant obese patients by affecting ER stress (Kars et al., 2010). Low dose of naltrexone treatment improved the function of epithelial barrier in IBD patients by reducing ER stress (Lie et al., 2018). These studies further demonstrate the feasibility of ER stress inhibitors as a treatment for parasitic diseases. In addition, the selectively target molecules of ER stress are more likely to be used, such as the inhibitors of ER stress molecules that play a key role in the survival and development of parasites (e.g., GSK2606414, PERK inhibitor) (Zhang et al., 2017), or the molecules of ER stress of parasites that are different from the host genes (e.g., GRP78 of *P. falciparum*) (Chen et al., 2018), or the parasites that are more sensitive to ER stress inducers than the host (e.g., DTT-induced ER stress in *Leishmania*) (Gosline et al., 2011). Moreover, because ER stress is involved in drug resistance, the inhibitors of ER stress molecules can be used in combination with anti-parasite drugs, such as ER stress inhibitors or PI3P tubules/vesicles inhibitors that may be used in patients with artemisinin resistance (Mok et al., 2015; Bhattacharjee et al., 2018).

**TABLE 1:** Potential drug targets of UPR for treatment of parasitosis.

**Inhibitors**	**Target molecules**	**Function/mechanism of inhibitor**	**Effects**	**References**
2-aminopurine	PERK-eIF2α- CHOP pathway	Inhibiting eIF2α phosphorylation and its downstream signaling	Alleviating *T. cruzi* infection induced-heart damage	Ayyappan et al., 2019
TUDCA/Taurine	CHOP-cleaved caspase-12 pathway; GRP78-CHOP pathway	Inhibiting ER stress induced cell apoptosis	Alleviating *T. gondii* infection induced-*Toxoplasma* encephalitis; Alleviating *S. japonicum* infection induced-hepatic granuloma and fibrosis	Wang et al., 2014; Yu et al., 2016
ROP18 inhibitors	ROP18 of *T. gondii*	Inhibiting *T. gondii* infection induced-nerve cell apoptosis by ER stress pathway	Alleviating *Toxoplasma* encephalitis	Wan et al., 2015; Tang et al., 2017; An et al., 2018
GSK2606414	PERK homolog PK4 of *Plasmodium;* PERK homolog TgIF2K-A of *Toxoplasma*	Inhibiting the activation of PK4 and phosphorylation of eIF2α; inhibiting the lytic cycle of tachyzoites	Alleviating the symptoms of malaria, preventing the recurrence of *Plasmodium* and inhibit the transmission of this disease; Inhibiting the invasion, replication and differentiation of *T. gondii*	Zhang et al., 2017; Augusto et al., 2018
apoptozole	GRP78 of *P. falciparum*	Inhibiting GRP78 function	Leading to the death of chloroquine-sensitive and -resistant *P. falciparum* strains	Chen et al., 2018
ER stress inducer	PERK pathway of *Leishmania*; eIF2α of *Plasmodium*; Bip of *T. brucei;* PERK pathway of *T. gondii;* TgIF2α of *T. gondii;* Bip of *Leishmania major*	Inducing eIF2α phosphorylation; Inducing eIF2α phosphorylation of *Plasmodium;* Increasing the expression of Bip of *T. brucei;* Inducing eIF2α phosphorylation of *T. gondii;* Inducing the phosphorylation of TgIF2α; Increasing the expression of Bip of *Leishmania major*	Kill parasites (The parasite is more susceptible to ER stress than host due to the mere presence of the PERK pathway); Participating in the formation of *P. falciparum* gametophytes and the conversion of the *P. berghei;* Inducing programmed cell death of *T. brucei;* Inducing apoptosis or autophagy of *T. gondii;* Inhibiting the differentiation of *T. gondii* from tachyzoite to bradyzoite cysts; Inducing the apoptosis of *Leishmania major*	Narasimhan et al., 2008; Goldshmidt et al., 2010; Dolai et al., 2011; Gosline et al., 2011; Chaubey et al., 2014; Duran-Bedolla et al., 2017; Nguyen et al., 2017; Hao et al., 2019
TbeIF2K2 inhibitors	PERK homolog TbeIF2K2 of *T. brucei*	May suppress the function of sensing protein and regulating protein synthesis near flagellar pocket of *Trypanosome*	Inhibiting the survival of parasites	Gull, 2003; Moraes et al., 2007
PK3 activator	PERK homolog PK3 of *T. brucei*	Increasing ER stress-induced PCD	Lead to the death of *T. brucei*	Hope et al., 2014
Tc-eIF2α phosphorylation inhibitor	Tc-eIF2α of *T. cruzi*	Inhibiting the phosphorylation of Tc-eIF2α	Inhibiting the differentiation of non-infective epimastigotes into infective metacyclic trypomastigotes	Tonelli et al., 2011
TgIF2α phosphorylation inhibitor Salubrinal	TgIF2α of *T. gondii*	Inhibiting the phosphorylation of TgIF2α	Inhibiting the survival of *Toxoplasma* and decreasing virulence to host cell	Narasimhan et al., 2008; Joyce et al., 2010
Bortezomib	GRP78- IRE2/XBP1 pathway of protoscoleces of *E. granulosus*	Inducing ER stress and apoptosis	Reducing the viability of *E. granulosus*	Nicolao et al., 2017
PI3P tubules/vesicles inhibitor	PI3P tubules/vesicles of *Plasmodium*	Inhibiting the formation and diffusion of PI3P tubules/vesicles	Inhibiting UPR mediated artemisinin resistance	Mok et al., 2015; Bhattacharjee et al., 2018

*PERK, protein kinase RNA-like ER kinase; IRE1, inositol-requiring kinase/endoribonuclease 1; ATF6, activating transcription factor 6; ATF4, activating transcription factor 4; XBP1, X box-binding protein 1; CHOP, C/EBP-homologous protein; GRP78, Glucose regulated proteins 78; TUDCA, tauroursodeoxycholic acid; ROP18, rhoptry protein 18; eIF2α, α-subunit of eukaryotic translational initiation factor 2; PCD, programmed cell death; PI3P, phosphatidylinositol-3-phosphate.*

## Author Contributions

JS advocated writing this review, reviewed, edited, and approved its final version. MP collected literature and wrote the manuscript. FC collected and reviewed literature. ZW provided some suggestions for this review. All authors contributed to the article and approved the submitted version.

## Conflict of Interest

The authors declare that the research was conducted in the absence of any commercial or financial relationships that could be construed as a potential conflict of interest.

## References

[B1] Abhishek K. Das S. Kumar A. Kumar A. Kumar V. Saini S. (2018). *Leishmania donovani* induced unfolded protein response delays host cell apoptosis in PERK dependent manner. *PLoS Neglect. Trop. Dis.* 12:e0006646. 10.1371/journal.pntd.0006646 30036391 PMC6081962

[B2] Acosta-Alvear D. Zhou Y. Blais A. Tsikitis M. Lents N. H. Arias C. (2007). XBP1 controls diverse cell type- and condition-specific transcriptional regulatory networks. *Mol. Cell* 27 53–66. 17612490 10.1016/j.molcel.2007.06.011

[B3] Agrawal S. Chung D. W. D. Ponts N. van Dooren G. G. Prudhomme J. Brooks C. F. (2013). An apicoplast localized Ubiquitylation system is required for the import of nuclear-encoded plastid proteins. *PLoS Pathog.* 9:e1003426. 10.1371/journal.ppat.1003426 23785288 PMC3681736

[B4] Agrawal S. van Dooren G. G. Beatty W. L. Striepen B. (2009). Genetic evidence that an endosymbiont-derived endoplasmic reticulum-associated protein degradation (ERAD) system functions in import of apicoplast proteins. *J. Biol. Chem.* 284 33683–33691. 10.1074/jbc.M109.044024 19808683 PMC2785210

[B5] An R. Tang Y. Chen L. Cai H. Lai D.-H. Liu K. (2018). Encephalitis is mediated by ROP18 of, a severe pathogen in AIDS patients. *Proc.Natl. Acad. Sci. U.S.A.* 115 E5344–E5352. 10.1073/pnas.1801118115 29784816 PMC6003310

[B6] Anand S. S. Babu P. P. (2013). Endoplasmic reticulum stress and neurodegeneration in experimental cerebral malaria. *Neuro Signals* 21:79. 10.1159/000336970 22584375

[B7] Augusto L. Martynowicz J. Amin P. H. Alakhras N. S. Kaplan M. H. Wek R. C. (2020). *Toxoplasma gondii* Co-opts the unfolded protein response to enhance migration and dissemination of infected host cells. *mBio* 11:e00915-20. 10.1128/mBio.00915-20 32636244 PMC7343987

[B8] Augusto L. Martynowicz J. Staschke K. A. Wek R. C. Sullivan W. J. (2018). Effects of PERK eIF2α kinase inhibitor against *Toxoplasma gondii*. *Antimicrob. Agents Chemother.* 62:e001442-18. 10.1128/AAC.01442-18 30181373 PMC6201070

[B9] Axten J. M. Medina J. R. Feng Y. Shu A. Romeril S. P. Grant S. W. (2012). Discovery of 7-methyl-5-(1-{[3-(trifluoromethyl)phenyl]acetyl}-2,3-dihydro-1H-indol-5-yl)-7H-pyrrolo[2,3-d]pyrimidin-4-amine (GSK2606414), a potent and selective first-in-class inhibitor of protein kinase R (PKR)-like endoplasmic reticulum kinase (PERK). *J. Med. Chem.* 55 7193–7207. 10.1021/jm300713s 22827572

[B10] Ayyappan J. P. Lizardo K. Wang S. Yurkow E. Nagajyothi J. F. (2019). Inhibition of ER stress by 2-aminopurine treatment modulates cardiomyopathy in a murine chronic chagas disease model. *Biomol. Ther.* 27 386–394. 10.4062/biomolther.2018.193 30879276 PMC6609105

[B11] Bertolotti A. Zhang Y. Hendershot L. M. Harding H. P. Ron D. (2000). Dynamic interaction of BiP and ER stress transducers in the unfolded-protein response. *Nat. Cell Biol.* 2 326–332. 10854322 10.1038/35014014

[B12] Bhattacharjee S. Coppens I. Mbengue A. Suresh N. Ghorbal M. Slouka Z. (2018). Remodeling of the malaria parasite and host human red cell by vesicle amplification that induces artemisinin resistance. *Blood* 131 1234–1247. 10.1182/blood-2017-11-814665 29363540 PMC5855022

[B13] Black M. W. Boothroyd J. C. (2000). Lytic cycle of *Toxoplasma gondii*. *Microbiol. Mol. Biol. Rev.* 64 607–623. 10974128 10.1128/mmbr.64.3.607-623.2000 PMC99006

[B14] Bridgford J. L. Xie S. C. Cobbold S. A. Pasaje C. F. A. Herrmann S. Yang T. (2018). Artemisinin kills malaria parasites by damaging proteins and inhibiting the proteasome. *Nat. Commun.* 9:3801. 10.1038/s41467-018-06221-1 30228310 PMC6143634

[B15] Bukau B. Weissman J. Horwich A. (2006). Molecular chaperones and protein quality control. *Cell* 125 443–451. 16678092 10.1016/j.cell.2006.04.014

[B16] Calfon M. Zeng H. Urano F. Till J. H. Hubbard S. R. Harding H. P. (2002). IRE1 couples endoplasmic reticulum load to secretory capacity by processing the XBP-1 mRNA. *Nature* 415 92–96. 11780124 10.1038/415092a

[B17] Chaubey S. Grover M. Tatu U. (2014). Endoplasmic reticulum stress triggers gametocytogenesis in the malaria parasite. *J. Biol. Chem.* 289 16662–16674. 10.1074/jbc.M114.551549 24755215 PMC4059112

[B18] Chen Y. Murillo-Solano C. Kirkpatrick M. G. Antoshchenko T. Park H. W. Pizarro J. C. (2018). Repurposing drugs to target the malaria parasite unfolding protein response. *Sci. Rep.* 8:10333. 10.1038/s41598-018-28608-2 29985421 PMC6037779

[B19] Chow C. Cloutier S. Dumas C. Chou M.-N. Papadopoulou B. (2011). Promastigote to amastigote differentiation of *Leishmania* is markedly delayed in the absence of PERK eIF2alpha kinase-dependent eIF2alpha phosphorylation. *Cell. Microbiol.* 13 1059–1077. 10.1111/j.1462-5822.2011.01602.x 21624030

[B20] Chung D.-W. D. Ponts N. Prudhomme J. Rodrigues E. M. Le Roch K. G. (2012). Characterization of the ubiquitylating components of the human malaria parasite’s protein degradation pathway. *PLoS One* 7:e43477. 10.1371/journal.pone.0043477 22912882 PMC3422240

[B21] Dias-Teixeira K. L. Calegari-Silva T. C. dos Santos G. R. R. M. Vitorino Dos Santos J. Lima C. Medina J. M. (2016). The integrated endoplasmic reticulum stress response in *Leishmania amazonensis* macrophage infection: the role of X-box binding protein 1 transcription factor. *FASEB J.* 30 1557–1565. 10.1096/fj.15-281550 26678450 PMC7163978

[B22] Dias-Teixeira K. L. Calegari-Silva T. C. Medina J. M. Vivarini A. C. Cavalcanti A. Teteo N. (2017). Emerging role for the PERK/eIF2alpha/ATF4 in human Cutaneous *Leishmaniasis*. *Sci. Rep.* 7:17074. 10.1038/s41598-017-17252-x 29213084 PMC5719050

[B23] Dolai S. Adak S. (2014). Endoplasmic reticulum stress responses in *Leishmania*. *Mol. Biochem. Parasitol.* 197 1–8. 10.1016/j.molbiopara.2014.09.002 25224909

[B24] Dolai S. Pal S. Yadav R. K. Adak S. (2011). Endoplasmic reticulum stress-induced apoptosis in *Leishmania* through Ca2+-dependent and caspase-independent mechanism. *J. Biol. Chem.* 286 13638–13646. 10.1074/jbc.M110.201889 21330370 PMC3075708

[B25] Dondorp A. M. Nosten F. Yi P. Das D. Phyo A. P. Tarning J. (2009). Artemisinin resistance in *Plasmodium falciparum* malaria. *New Engl. J. Med.* 361 455–467. 10.1056/NEJMoa0808859 19641202 PMC3495232

[B26] Duan M. Yang Y. Peng S. Liu X. Zhong J. Guo Y. (2019). C/EBP homologous protein (CHOP) activates macrophages and promotes liver fibrosis in *Schistosoma japonicum*-infected mice. *J. Immunol. Res.* 2019:5148575. 10.1155/2019/5148575 31886304 PMC6914929

[B27] Duran-Bedolla J. Tellez-Sosa J. Valdovinos-Torres H. Pavon N. Buelna-Chontal M. Tello-Lopez A. T. (2017). Cellular stress associated with the differentiation of *Plasmodium berghei* ookinetes. *Biochem. Cell Biol.* 95 310–317. 10.1139/bcb-2016-0028 28177775

[B28] Fallon P. G. Doenhoff M. J. (1994). Drug-resistant schistosomiasis: resistance to praziquantel and oxamniquine induced in *Schistosoma mansoni* in mice is drug specific. *Am. J. Trop. Med. Hyg.* 51 83–88. 8059919 10.4269/ajtmh.1994.51.83

[B29] Galluzzi L. Diotallevi A. De Santi M. Ceccarelli M. Vitale F. Brandi G. (2016). *Leishmania infantum* induces mild unfolded protein response in infected macrophages. *PLoS One* 11:e0168339. 10.1371/journal.pone.0168339 27978534 PMC5158320

[B30] Galluzzi L. Diotallevi A. Magnani M. (2017). Endoplasmic reticulum stress and unfolded protein response in infection by intracellular parasites. *Future Sci. OA* 3:FSO198. 10.4155/fsoa-2017-0020 28883998 PMC5583660

[B31] Gass J. N. Gifford N. M. Brewer J. W. (2002). Activation of an unfolded protein response during differentiation of antibody-secreting B cells. *J. Biol. Chem.* 277 49047–49054. 12374812 10.1074/jbc.M205011200

[B32] Goldshmidt H. Matas D. Kabi A. Carmi S. Hope R. Michaeli S. (2010). Persistent ER stress induces the spliced leader RNA silencing pathway (SLS), leading to programmed cell death in *Trypanosoma brucei*. *PLoS Pathog.* 6:e1000731. 10.1371/journal.ppat.1000731 20107599 PMC2809764

[B33] Gosline S. J. C. Nascimento M. McCall L.-I. Zilberstein D. Thomas D. Y. Matlashewski G. (2011). Intracellular eukaryotic parasites have a distinct unfolded protein response. *PLoS One* 6:e19118. 10.1371/journal.pone.0019118 21559456 PMC3084755

[B34] Grootjans J. Kaser A. Kaufman R. J. Blumberg R. S. (2016). The unfolded protein response in immunity and inflammation. *Nat. Rev. Immunol.* 16 469–484. 10.1038/nri.2016.62 27346803 PMC5310224

[B35] Gull K. (2003). Host-parasite interactions and *trypanosome* morphogenesis: a flagellar pocketful of goodies. *Curr. Opin. Microbiol.* 6 365–370. 12941406 10.1016/s1369-5274(03)00092-4

[B36] Haldar K. Bhattacharjee S. Safeukui I. (2018). Drug resistance in *Plasmodium*. *Nat. Rev. Microbiol.* 16 156–170. 10.1038/nrmicro.2017.161 29355852 PMC6371404

[B37] Hao P. Cui X. Liu J. Li M. Fu Y. Liu Q. (2019). Identification and characterization of stearoyl-CoA desaturase in *Toxoplasma gondii*. *Acta Biochim. Biophys. Sinica* 51 615–626. 10.1093/abbs/gmz040 31139819 PMC6574064

[B38] Harbut M. B. Patel B. A. Yeung B. K. McNamara C. W. Bright A. T. Ballard J. (2012). Targeting the ERAD pathway via inhibition of signal peptide peptidase for antiparasitic therapeutic design. *Proc. Natl. Acad. Sci. U.S.A.* 109 21486–21491. 10.1073/pnas.1216016110 23236186 PMC3535666

[B39] Haze K. Yoshida H. Yanagi H. Yura T. Mori K. (1999). Mammalian transcription factor ATF6 is synthesized as a transmembrane protein and activated by proteolysis in response to endoplasmic reticulum stress. *Mol. Biol. Cell* 10 3787–3799. 10564271 10.1091/mbc.10.11.3787 PMC25679

[B40] Hendrick H. M. Welter B. H. Hapstack M. A. Sykes S. E. Sullivan W. J. Temesvari L. A. (2016). Phosphorylation of eukaryotic initiation factor-2α during Stress and encystation in *Entamoeba* Species. *PLoS Pathog.* 12:e1006085. 10.1371/journal.ppat.1006085 27930733 PMC5179133

[B41] Hetz C. Chevet E. Harding H. P. (2013). Targeting the unfolded protein response in disease. *Nat. Rev. Drug Discov.* 12 703–719. 10.1038/nrd3976 23989796

[B42] Hope R. Ben-Mayor E. Friedman N. Voloshin K. Biswas D. Matas D. (2014). Phosphorylation of the TATA-binding protein activates the spliced leader silencing pathway in *Trypanosoma brucei*. *Sci. Signal.* 7:ra85. 10.1126/scisignal.2005234 25185157

[B43] Hwang J. Qi L. (2018). Quality control in the endoplasmic reticulum: crosstalk between ERAD and UPR pathways. *Trends Biochem. Sci.* 43 593–605. 10.1016/j.tibs.2018.06.005 30056836 PMC6327314

[B44] Inacio P. Zuzarte-Luis V. Ruivo M. T. Falkard B. Nagaraj N. Rooijers K. (2015). Parasite-induced ER stress response in hepatocytes facilitates *Plasmodium* liver stage infection. *EMBO Rep.* 16 955–964. 10.15252/embr.201439979 26113366 PMC4552488

[B45] Joyce B. R. Queener S. F. Wek R. C. Sullivan W. J. (2010). Phosphorylation of eukaryotic initiation factor-2{alpha} promotes the extracellular survival of obligate intracellular parasite *Toxoplasma gondii*. *Proc. Natl. Acad. Sci. U.S.A.* 107 17200–17205. 10.1073/pnas.1007610107 20855600 PMC2951449

[B46] Joyce B. R. Tampaki Z. Kim K. Wek R. C. Sullivan W. J. (2013). The unfolded protein response in the protozoan parasite *Toxoplasma gondii* features translational and transcriptional control. *Eukaryot. Cell* 12 979–989. 10.1128/EC.00021-13 23666622 PMC3697469

[B47] Kars M. Yang L. Gregor M. F. Mohammed B. S. Pietka T. A. Finck B. N. (2010). Tauroursodeoxycholic Acid may improve liver and muscle but not adipose tissue insulin sensitivity in obese men and women. *Diabetes* 59 1899–1905. 10.2337/db10-0308 20522594 PMC2911053

[B48] Konrad C. Wek R. C. Sullivan W. J. (2014). GCN2-like eIF2α kinase manages the amino acid starvation response in *Toxoplasma gondii*. *Intern. J. Parasitol.* 44 139–146. 10.1016/j.ijpara.2013.08.005 24126185 PMC3946947

[B49] Kumari R. Gupta P. Tiwari S. (2018). Ubc7/Ube2g2 ortholog in *Entamoeba histolytica*: connection with the plasma membrane and phagocytosis. *Parasitol. Res.* 117 1599–1611. 10.1007/s00436-018-5842-6 29594345

[B50] Lazaridis K. N. Gores G. J. Lindor K. D. (2001). Ursodeoxycholic acid ‘mechanisms of action and clinical use in hepatobiliary disorders’. *J. Hepatol.* 35 134–146. 11495032 10.1016/s0168-8278(01)00092-7

[B51] Li J. Tang G. Qin W. Yang R. Ma R. Ma B. (2018). Toxic effects of arsenic trioxide on *Echinococcus granulosus* protoscoleces through ROS production, and Ca2+-ER stress-dependent apoptosis. *Acta Biochimi. Biophys. Sinica* 50 579–585. 10.1093/abbs/gmy041 29684096

[B52] Li P. Fu D. Sheng Q. Yu S. Bao X. Lv Z. (2019). TUDCA attenuates intestinal injury and inhibits endoplasmic reticulum stress-mediated intestinal cell apoptosis in necrotizing enterocolitis. *Intern. Immunopharmacol.* 74:105665. 10.1016/j.intimp.2019.05.050 31254957

[B53] Lie M. R. K. L. van der Giessen J. Fuhler G. M. de Lima A. Peppelenbosch M. P. van der Ent C. (2018). Low dose Naltrexone for induction of remission in inflammatory bowel disease patients. *J. Transl. Med.* 16:55. 10.1186/s12967-018-1427-5 29523156 PMC5845217

[B54] Liu F. Cui Y. Ge P. Luan J. Zhou X. Han J. (2015). Tauroursodeoxycholic acid attenuates inorganic phosphate-induced osteoblastic differentiation and mineralization in NIH3T3 fibroblasts by inhibiting the ER stress response PERK-eIF2α-ATF4 pathway. *Drug Discov. Therap.* 9 38–44. 25788050 10.5582/ddt.2015.01008

[B55] Loos J. A. Nicolao M. C. Cumino A. C. (2018). Metformin promotes autophagy in *Echinococcus granulosus* larval stage. *Mol. Biochem. Parasitol.* 224 61–70. 10.1016/j.molbiopara.2018.07.003 30017657

[B56] Ma Y. Hendershot L. M. (2003). Delineation of a negative feedback regulatory loop that controls protein translation during endoplasmic reticulum stress. *J. Biol. Chem.* 278 34864–34873. 12840028 10.1074/jbc.M301107200

[B57] Malo A. Krüger B. Seyhun E. Schäfer C. Hoffmann R. T. Göke B. (2010). Tauroursodeoxycholic acid reduces endoplasmic reticulum stress, trypsin activation, and acinar cell apoptosis while increasing secretion in rat pancreatic acini. *Am. J. Physiol. Gastrointest. Liver Physiol.* 299 G877–G886. 10.1152/ajpgi.00423.2009 20671193

[B58] Messias Sandes J. Nascimento Moura D. M. Divina da Silva Santiago M. Barbosa de Lima G. Cabral Filho P. E. da Cunha Goncalves de Albuquerque S. (2019). The effects of endoplasmic reticulum stressors, tunicamycin and dithiothreitol on *Trypanosoma cruzi*. *Exp. Cell Res.* 383:111560. 10.1016/j.yexcr.2019.111560 31437457

[B59] Möhrle J. J. Zhao Y. Wernli B. Franklin R. M. Kappes B. (1997). Molecular cloning, characterization and localization of PfPK4, an eIF-2alpha kinase-related enzyme from the malarial parasite *Plasmodium falciparum*. *Biochem. J.* 328(Pt 2), 677–687. 9371731 10.1042/bj3280677 PMC1218971

[B60] Mok S. Ashley E. A. Ferreira P. E. Zhu L. Lin Z. Yeo T. (2015). Drug resistance. Population transcriptomics of human malaria parasites reveals the mechanism of artemisinin resistance. *Science* 347 431–435. 10.1126/science.1260403 25502316 PMC5642863

[B61] Moraes M. C. S. Jesus T. C. L. Hashimoto N. N. Dey M. Schwartz K. J. Alves V. S. (2007). Novel membrane-bound eIF2alpha kinase in the flagellar pocket of *Trypanosoma brucei*. *Eukaryot. Cell* 6 1979–1991. 17873083 10.1128/EC.00249-07 PMC2168417

[B62] Narasimhan J. Joyce B. R. Naguleswaran A. Smith A. T. Livingston M. R. Dixon S. E. (2008). Translation regulation by eukaryotic initiation factor-2 kinases in the development of latent cysts in *Toxoplasma gondii*. *J. Biol. Chem.* 283 16591–16601. 10.1074/jbc.M800681200 18420584 PMC2423249

[B63] Nguyen H. M. Berry L. Sullivan W. J. Besteiro S. (2017). Autophagy participates in the unfolded protein response in *Toxoplasma gondii*. *FEMS Microbiol. Lett.* 364:fnx153. 10.1093/femsle/fnx153 28859319 PMC5827624

[B64] Nicolao M. C. Loos J. A. Rodriguez Rodrigues C. Beas V. Cumino A. C. (2017). Bortezomib initiates endoplasmic reticulum stress, elicits autophagy and death in *Echinococcus granulosus* larval stage. *PLoS One* 12:e0181528. 10.1371/journal.pone.0181528 28817601 PMC5560652

[B65] Novoa I. Zeng H. Harding H. P. Ron D. (2001). Feedback inhibition of the unfolded protein response by GADD34-mediated dephosphorylation of eIF2alpha. *J. Cell Biol.* 153 1011–1022. 11381086 10.1083/jcb.153.5.1011 PMC2174339

[B66] Pahl H. L. Baeuerle P. A. (1995). A novel signal transduction pathway from the endoplasmic reticulum to the nucleus is mediated by transcription factor NF-kappa B. *EMBO J.* 14 2580–2588. 7781611 10.1002/j.1460-2075.1995.tb07256.x PMC398372

[B67] Pineda E. Perdomo D. (2017). *Entamoeba histolytica* under oxidative stress: what countermeasure mechanisms are in place? *Cells* 6:44. 10.3390/cells6040044 29160807 PMC5755502

[B68] Poncet A. F. Bosteels V. Hoffmann E. Chehade S. Rennen S. Huot L. (2021). The UPR sensor IRE1α promotes dendritic cell responses to control *Toxoplasma gondii* infection. *EMBO Rep.* 22:e49617. 10.15252/embr.201949617 33586853 PMC7926260

[B69] Ron D. Walter P. (2007). Signal integration in the endoplasmic reticulum unfolded protein response. *Nat. Rev. Mol. Cell Biol.* 8 519–529. 17565364 10.1038/nrm2199

[B70] Santi-Rocca J. Smith S. Weber C. Pineda E. Hon C.-C. Saavedra E. (2012). Endoplasmic reticulum stress-sensing mechanism is activated in *Entamoeba histolytica* upon treatment with nitric oxide. *PLoS One* 7:e31777. 10.1371/journal.pone.0031777 22384074 PMC3286455

[B71] Shen J. Chen X. Hendershot L. Prywes R. (2002). ER stress regulation of ATF6 localization by dissociation of BiP/GRP78 binding and unmasking of Golgi localization signals. *Dev. Cell* 3 99–111. 12110171 10.1016/s1534-5807(02)00203-4

[B72] Spork S. Hiss J. A. Mandel K. Sommer M. Kooij T. W. A. Chu T. (2009). An unusual ERAD-Like complex is targeted to the apicoplast of *Plasmodium falciparum*. *Eukaryot. Cell* 8 1134–1145. 10.1128/Ec.00083-09 19502583 PMC2725561

[B73] Sullivan W. J. Narasimhan J. Bhatti M. M. Wek R. C. (2004). Parasite-specific eIF2 (eukaryotic initiation factor-2) kinase required for stress-induced translation control. *Biochem. J.* 380(Pt 2), 523–531. 14989696 10.1042/BJ20040262 PMC1224182

[B74] Tang Y. W. Zheng M. J. An R. Chen L. J. Gong L. L. Cai H. J. (2017). Proteasomal degradation of *T. gondii* ROP18 requires Derlin2. *Acta Trop.* 174 106–113. 10.1016/j.actatropica.2017.06.027 28669563

[B75] Tiengwe C. Muratore K. A. Bangs J. D. (2016). Surface proteins, ERAD and antigenic variation in *Trypanosoma brucei*. *Cell. Microbiol.* 18 1673–1688. 10.1111/cmi.12605 27110662 PMC5575760

[B76] Tonelli R. R. Augusto Lda S. Castilho B. A. Schenkman S. (2011). Protein synthesis attenuation by phosphorylation of eIF2alpha is required for the differentiation of *Trypanosoma cruzi* into infective forms. *PLoS One* 6:e27904. 10.1371/journal.pone.0027904 22114724 PMC3218062

[B77] Tsuru A. Imai Y. Saito M. Kohno K. (2016). Novel mechanism of enhancing IRE1α-XBP1 signalling via the PERK-ATF4 pathway. *Sci. Rep.* 6:24217. 10.1038/srep24217 27052593 PMC4823713

[B78] Walters H. A. Welter B. H. Sullivan W. J. Temesvari L. A. (2019). Phosphorylation of eukaryotic initiation factor-2α in response to endoplasmic reticulum and nitrosative stress in the human protozoan parasite, *Entamoeba histolytica*. *Mol. Biochem. Parasitol.* 234:111223. 10.1016/j.molbiopara.2019.111223 31568804 PMC6886254

[B79] Wan L. Gong L. Wang W. An R. Zheng M. Jiang Z. (2015). *T. gondii* rhoptry protein ROP18 induces apoptosis of neural cells via endoplasmic reticulum stress pathway. *Parasit. Vect.* 8:554. 10.1186/s13071-015-1103-z 26489755 PMC4618732

[B80] Wang T. Zhou J. Gan X. Wang H. Ding X. Chen L. (2014). *Toxoplasma gondii* induce apoptosis of neural stem cells via endoplasmic reticulum stress pathway. *Parasitology* 141 988–995. 10.1017/S0031182014000183 24612639

[B81] Ward P. Equinet L. Packer J. Doerig C. (2004). Protein kinases of the human malaria parasite *Plasmodium falciparum*: the kinome of a divergent eukaryote. *BMC Genom.* 5:79. 10.1186/1471-2164-5-79 15479470 PMC526369

[B82] Yoshida H. Matsui T. Yamamoto A. Okada T. Mori K. (2001). XBP1 mRNA is induced by ATF6 and spliced by IRE1 in response to ER stress to produce a highly active transcription factor. *Cell* 107 881–891. 11779464 10.1016/s0092-8674(01)00611-0

[B83] Yu Y.-R. Deng M.-J. Lu W.-W. Zhang J.-S. Jia M.-Z. Huang J. (2014). Endoplasmic reticulum stress-mediated apoptosis is activated in intestines of mice with *Trichinella spiralis* infection. *Exper. Parasitol.* 145 1–6. 10.1016/j.exppara.2014.06.017 24996067

[B84] Yu Y.-R. Ni X.-Q. Huang J. Zhu Y.-H. Qi Y.-F. (2016). Taurine drinking ameliorates hepatic granuloma and fibrosis in mice infected with *Schistosoma japonicum*. *Intern. J. Parasitol. Drugs Drug Resist.* 6 35–43. 10.1016/j.ijpddr.2016.01.003 27054062 PMC4805782

[B85] Zhang M. Gallego-Delgado J. Fernandez-Arias C. Waters N. C. Rodriguez A. Tsuji M. (2017). Inhibiting the *Plasmodium* eIF2alpha Kinase PK4 prevents artemisinin-induced latency. *Cell Host Microb.* 22 766–776.e764. 10.1016/j.chom.2017.11.005 29241041 PMC5869688

[B86] Zhang M. Mishra S. Sakthivel R. Rojas M. Ranjan R. Sullivan W. J. (2012). PK4, a eukaryotic initiation factor 2 (eIF2) kinase, is essential for the development of the erythrocytic cycle of *Plasmodium*. *Proc. Natl. Acad. Sci. U.S.A.* 109 3956–3961. 10.1073/pnas.1121567109 22355110 PMC3309761

[B87] Zhang X. An T. Pham K. T. M. Lun Z.-R. Li Z. (2019). Functional analyses of cytokinesis regulators in bloodstream stage *Trypanosoma brucei* parasites identify functions and regulations specific to the life cycle stage. *mSphere* 4:e0199-19. 10.1128/mSphere.00199-19 31043517 PMC6495339

[B88] Zhou J. Gan X. Wang Y. Zhang X. Ding X. Chen L. (2015). *Toxoplasma gondii* prevalent in China induce weaker apoptosis of neural stem cells C17.2 via endoplasmic reticulum stress (ERS) signaling pathways. *Parasit. Vect.* 8:73. 10.1186/s13071-015-0670-3 25649541 PMC4322664

[B89] Zuzarte-Luís V. Mota M. M. (2018). Parasite sensing of host nutrients and environmental cues. *Cell Host Microb.* 23 749–758. 10.1016/j.chom.2018.05.018 29902440

